# Genome-resolved metaproteogenomic and nanosolid characterization of an inactive vent chimney densely colonized by enigmatic DPANN archaea

**DOI:** 10.1093/ismejo/wrae207

**Published:** 2024-11-05

**Authors:** Hinako Takamiya, Mariko Kouduka, Shingo Kato, Hiroki Suga, Masaki Oura, Tadashi Yokoyama, Michio Suzuki, Masaru Mori, Akio Kanai, Yohey Suzuki

**Affiliations:** Department of Earth and Planetary Science, The University of Tokyo, Bunkyo-ku, Tokyo, Japan; Department of Earth and Planetary Science, The University of Tokyo, Bunkyo-ku, Tokyo, Japan; Japan Collection of Microorganisms (JCM), RIKEN BioResource Research Center, Tsukuba, Ibaraki, Japan; Submarine Resources Research Center, Japan Agency for Marine-Earth Science and Technology (JAMSTEC), 2-15, Natsushima-cho, Yokosuka-city, Kanagawa 237-0061, Japan; Spectroscopy Division, Japan Synchrotron Radiation Research Institute, Sayo-gun, Hyogo, Japan; Graduate School of Advanced Science and Engineering, Hiroshima University, Kagamiyama, Higashi-Hiroshima, Hiroshima, Japan; Soft X-ray Spectroscopy Instrumentation Team, RIKEN SPring-8 Center, Sayo-gun, Hyogo, Japan; Graduate School of Advanced Science and Engineering, Hiroshima University, Kagamiyama, Higashi-Hiroshima, Hiroshima, Japan; Department of Applied Biological Chemistry, The University of Tokyo, Yayoi, Bunkyo-ku, Tokyo, Japan; Institute for Advanced Biosciences, Keio University, Nipponkoku, Daihoji, Tsuruoka, Yamagata, Japan; Institute for Advanced Biosciences, Keio University, Nipponkoku, Daihoji, Tsuruoka, Yamagata, Japan; Department of Earth and Planetary Science, The University of Tokyo, Bunkyo-ku, Tokyo, Japan

**Keywords:** Pacearchaeota, Nitrosococcaceae, Hydrothermarchaeota, seafloor metal sulfide deposits, deep-sea hydrothermal vent

## Abstract

Recent successes in the cultivation of DPANN archaea with their hosts have demonstrated an episymbiotic lifestyle, whereas the lifestyle of DPANN archaea in natural habitats is largely unknown. A free-living lifestyle is speculated in oxygen-deprived fluids circulated through rock media, where apparent hosts of DPANN archaea are lacking. Alternatively, DPANN archaea may be detached from their hosts and/or rock surfaces. To understand the ecology of rock-hosted DPANN archaea, rocks rather than fluids should be directly characterized. Here, we investigated a deep-sea hydrothermal vent chimney without fluid venting where our previous study revealed the high proportion of *Pacearchaeota*, one of the widespread and enigmatic lineages of DPANN archaea. Using spectroscopic methods with submicron soft X-ray and infrared beams, the microbial habitat was specified to be silica-filled pores in the inner chimney wall comprising chalcopyrite. Metagenomic analysis of the inner wall revealed the lack of biosynthetic genes for nucleotides, amino acids, cofactors, and lipids in the *Pacearchaeota* genomes. Genome-resolved metaproteomic analysis clarified the co-occurrence of a novel thermophilic lineage actively fixing carbon and nitrogen and thermophilic archaea in the inner chimney wall. We infer that the shift in metabolically active microbial populations from the thermophiles to the mesophilic DPANN archaea occurs after the termination of fluid venting. The infilling of mineral pores by hydrothermal silica deposition might be a preferred environmental factor for the colonization of free-living *Pacearchaeota* with ultrasmall cells depending on metabolites synthesized by the co-occurring thermophiles during fluid venting.

## Introduction

The archaeal superphylum DPANN (originally proposed by five phyla: *Diapherotrites, Parvarchaeota, Aenigmarchaeota, Nanohaloarchaeota*, and *Nanoarchaeota*) was first recognized by single-cell genomics [1]. Complete and near-complete DPANN genomes, including additional major groups such as *Micraarcheota, Pacearcheota*, and *Woesearchaeta*, are obtained by metagenomic assembly and binning into metagenome-assembled genomes (MAGs) [2–4]. Based on comparative genomic analysis, DPANN archaea have small genomes encoding a minimum of metabolic enzymes [2, 5]. Thus, DPANN archaea are thought to depend on other microbes for most metabolites. By direct cell observations, along with phylogenomic profiling, DPANN archaea have ultrasmall cells (<200 nm in cell diameter) attached to host archaea cells in fluid samples from cocultures [6–10], acid mine drainages [3, 11, 12], and shallow and deep aquifers [13, 14]. However, recent single-cell genomic and metagenomic studies have suggested that DPANN archaea may also belong to complex microbial communities without symbiotic lifestyles [15, 16].

Novel archaeal lineages formerly referred to as deep-sea hydrothermal vent euryarchaeota (DHVE) were first recognized by 16S rRNA gene amplicon analysis [17]. Genome-based taxonomic classification reveals that DHVE-3, DHVE-5, and DHVE-6 are affiliated with *Aenigmarchaeota, Woesearchaeota,* and *Pacearchaeota*, respectively [18]. In deep-sea hydrothermal fields, DPANN-affiliated MAGs are obtained from vent fluids [19], sediments [20], seafloor, and sub-seafloor metal sulfide deposits [21, 22]. The widespread occurrence of DPANN in deep-sea hydrothermal fields provides a unique opportunity to study biological and environmental factors controlling the growth of DPANN archaea in spatiotemporally fluctuating habitats.

16S rRNA gene amplicon analysis and genome-resolved metagenomic analysis have been intensively performed for metal sulfide chimneys in association with and without fluid venting over the past two decades. Based on datasets complied from globally distributed deep-sea hydrothermal vents, it is well established that actively venting chimneys are colonized by chemoautotrophic members of *Aquificae* and *Camplyobacteria*, which disappear markedly after the termination of fluid venting [23–25]. As for microbial communities in chimneys without fluid venting (hereafter called inactive chimneys), microbial populations are similar in geographically and mineralogically distinct chimneys, whereas their proportions vary temporally after the termination of fluid venting and spatially within chimney structures [23–31]. Recent studies using genome-resolved metagenomics in combination with single-cell level metabolic activity measurements or metaproteomics have unveiled dominant microbes actively fixing carbon and nitrogen in inactive chimneys such as *Nitrospirae* and/or *Gammaproteobacteria* [24. 32].

We have studied an inactive chimney from the Southern Mariana Trough. The chimney sample exhibited mineral zonation characterized by chalcopyrite (CuFeS_2_) in the inner massive wall and the mixture of chalcopyrite and iron sulfide minerals such as marcasite (FeS_2_) and pyrite (FeS_2_) in the outer porous wall [33, 34]. 16S rRNA gene amplicon analysis revealed the high proportion of DPANN archaea (30% of the total high-quality reads) in the inner wall. In contrast, the dominance of bacteria affiliated within Nitrospirae was revealed by 16S rRNA gene amplicon analysis and genome-resolved metagenomic analysis [24], indicating the preference of DPANN archaea to the inner chimney wall [33]. Nanosolid characterizations such as nanoscale secondary ion mass spectrometry and transmission electron microscopy have been applied to the inner chimney wall. As a result, ultra-small cells with diameters of <200 nm were successfully visualized in chalcopyrite grain boundaries. However, these techniques with high-spatial resolutions require the embedment of the chimney sample into resin for preserving fragile microstructural features, which causes organic contamination. In addition, highly localized sampling from 10 × 10 μm square regions by focused ion beam fabrication is needed for these analytical techniques. Thus, it remains unknown whether the microbial distribution is widespread throughout the inner wall structure.

To extend the spatial coverage for microbial detection and mineral identification, we applied synchrotron-based soft X-ray spectroscopy and single-cell-level infrared spectroscopy to the inner chimney sample sectioned without resin embedment. In this study, 16S rRNA gene amplicon analysis and genome-resolved metagenomic and metaproteomic analyses were targeted to characterize the metabolic and ecological features of DPANN and co-occurring microbial populations in the inner chimney wall. By synthesizing these microbiological and geochemical data, we illuminate the temporal shift in the chimney habitat by silica deposition and the co-existence of enigmatic DPANN archaea with chemolithoautotrophic thermophiles.

## Materials and methods

Sample collection and subsampling. A metal sulfide chimney sample was collected from an active hydrothermal vent field at the Pika site (12°55.15’N, 143°36.96′E) in the Southern Mariana Trough during the Japan Agency for Marine-Earth Science and Technology (JAMSTEC) Scientific Cruise NT12–24 of the R/V *Natsushima* (September in 2012). Using the manipulator arm of the remotely operated vehicle (ROV) Hyper-Dolphin, the metal sulfide chimney sample was placed in a container to isolate it from the surrounding seawater during transportation to the surface. It was visually confirmed that the chimney structure lacked hydrothermal fluid venting. For the subsampling of the inner chimney wall, the outer chimney wall which was flamed with a gas torch, was thoroughly removed using sterile chisels and spatulas. This treatment has been applied to remove the contaminated exterior from drilled rock cores [35, 36]. An intact portion of the inner wall was preserved with 3.7% formaldehyde in seawater onboard and then substituted with 50% ethanol in distilled, deionized water for spectroscopic characterization. A portion of the inner wall sample ground into powder using sterile mortar and pestles was frozen at −80°C for total cell counting, 16S rRNA gene sequence analysis, and metagenomic and metaproteomic analyses.

Image analysis of chalcopyrite grain boundaries for the estimation of permeability. A 20-μm-thick section previously characterized was subjected to image analysis [37]. The permeability *k* (m^2^) was predicted with good accuracy using the following equation, *k* = 8.5(*ϕr*^2^)^1.3^, where *ϕ* (dimensionless) denotes the fraction of pores open to the outside, and *r* (m) represents the radius of the narrowest portion of the pore penetrating through the sample (pore throat). Image analysis was performed using ImageJ [38]. Each of the two analyzed areas in the Supplementary Fig. 1 was first converted to grayscale and then binarized (white: chalcopyrite grain; black: grain boundary). When using the algorithms provided with ImageJ for binarization thresholding, *ϕ* was overestimated in many cases (*ϕ* = 0.46–0.60). Alternatively, the minimum value *ϕ* was estimated to be 0.15 when determined visually. Therefore, the range of *ϕ* was set to be 0.15–0.46. Although *r* represents the pore throat, it is difficult to determine this value accurately in a 2D image. Therefore, after extracting a relatively long connected path using ImageJ, *r* was visually estimated by assuming that the typical radii of the path (2–5.3 μm) can approximate *r*. Substituting *r* = 2 μm and *ϕ* = 0.15 for the minimum value of *k* and *r* = 5.3 μm and *ϕ* = 0.46 for the maximum value of *k* into the above equation yields *k* = 1 × 10^−15^–6 × 10^−14^ m^2^.

Spectroscopic characterizations of the chimney interior. To clarify the distributions of microbes and minerals, the intact portion of the chimney structure was cut into 3-mm-thick sections using a precision diamond wire saw (Meiwa Fosis Corporation DWS 3500P). As the surface of the thin section was smooth, no further polishing was required for subsequent spectroscopic characterizations.

Soft X-ray spectromicroscopy developed at the soft X-ray undulator beamline BL17SU of SPring-8 was used for mapping light to heavy elements (C, N, O, Al, Si, Fe, and Cu) at a submicron resolution [39]. The incident soft X-ray beam was focused using the Fresnel zone plate. For micro XRF, the energy and intensity of the fluorescent soft X-rays emitted from the sample were analyzed using a silicon drift detector. For simultaneous measurements of seven elements (C: 120–170 ch; N: 185–240 ch; O: 268–313 ch; Fe: 368–410 ch; Cu: 480–540 ch; Al: 767–843 ch; Si: 920–960 ch), an excitation energy of 2100 eV was used. To obtain XRF spectra to resolve a peak of N from that of O, the excitation energy was lowered to 475 eV.

A mIRage infrared microscope (Photothermal Spectroscopy Corp., Santa Barbara, USA) was used to acquire O-PTIR spectra at a submicron resolution in refection mode (Cassegrain 40 objective (0.78 NA)) with a continuous wave (CW) 532-nm laser as the probe beam. The pump beam consisting of a tunable QCL device (950–1800 cm^−1^; 2-cm^−1^ spectral resolution and 10 scans per spectrum) was used to obtain O-PTIR spectra over the mid-IR ranges. Co-cultured cells of Nanoarchaeota strain MJ1 and *Metallosphaera* sp. strain MJ1HA (JCM33617) and cultured cells of *Shewanella oneidensis* (ATCC 700550) were lyophilized, and O-PTIR spectra were obtained by mounting on disks made of CaF_2_.

Total cell count. The total number of cells in the chimney interior was measured using a direct count method with SYBR Green I. 0.3 g of the frozen powdered sample was suspended in 3 ml of phosphate-buffered saline solution. 100 μl of the suspension was diluted in 10 ml of phosphate-buffered saline solution and then sonicated for 30 s at 50 W. A 0.1-μm-pore-size, 25-mm-diameter polycarbonate filter (GVS Filter Technology) was used to collect the sonicated suspension. To stain the microbial cells, the filter was incubated in a TAE buffer containing SYBR Green I for 5 min at room temperature. The stained filter was briefly rinsed with deionized water and observed under epifluorescence using the Olympus BX51 microscope with the Olympus DP71 CCD camera. A 100× objective lens was used with immersion oil, and we used the Olympus U-MWB2 wideband blue fluorescence filter cube for excitation 460–490 nm and emission >520 nm.

DNA extraction and 16S rRNA gene amplicon analysis. Prokaryotic DNA was extracted from the frozen powdered chimney subsample using chimerical and physical disruption methods. For chemical disruption initially developed to dissolve amorphous silica from diatoms [40], the powdered chimney subsample was incubated at 65°C for 30 min in an alkaline solution consisting of 0.5-M NaOH and TE buffer (Nippon Gene Co.). After centrifugation at 5000 × g for 30 s, the supernatant was neutralized with 1-M Tris–HCl (pH 6.5; Nippon Gene Co.). After neutralization, the DNA-bearing solution (pH 7.0–7.5 in TE buffer) was stored at −4°C or − 20°C for longer storage. For physical disruption, a DNeasy PowerSoil Pro Kit (Qiagen) was used, according to the manufacturer’s instructions.

The 16S rRNA gene sequences were amplified by PCR using LA Taq polymerase (TaKaRa-Bio, Inc., Shiga, Japan) using the primers Uni530F and Uni907R [41]. The PCR was performed in a reaction mixture containing 0.1 μM oligonucleotide primer and ca. 0.1 ng/μl DNA template with 35 cycles of denaturation at 96°C for 20 s, annealing at 56°C for 45 s, and extension at 72°C for 120 s. The first PCR product was used for the second PCR step, which was run for 10 cycles with Illumina TruSeq P5 and Index-containing P7 adapters. After purification using a MinElute Gel Extraction Kit (Qiagen, Inv., Valencia, CA), 16S rRNA gene sequencing was performed using the Illumina MiSeq Reagent Kit v2 on a MiSeq System (Illumina, San Diego, United States). The paired-end sequence reads were demultiplexed, trimmed, quality filtered, and chimera removed using standard Illumina software. The screened reads were imported into Qiime2 version 2022.2 [42] and DADA2 version 1.5.2 [43]. For alignment and taxonomic affiliation of the representative sequence obtained, SILVA SSU Ref NR database version 138 [44] was used in the QIIME2 program. For contamination control, DNA extraction was performed without the addition of the subsample. This blank sample was subjected to DNA sequencing, and sequences obtained from the laboratory contamination were distinct from those obtained from the chimney sample.

Genome-resolved metagenomic analysis. We used the same genomic DNA extracted by the physical disruption method described above for shotgun library construction with a KAPA Hyper Prep kit for Illumina (KAPA Biosystems) [45]. The sequencing of the library was performed on an Illumina MiSeq platform (MiSeq PE300). To reconstruct MAGs, the Read_QC module included in MetaWRAP v.1.3.2 [46] was used to trim and filter reads from the library. SPAdes version 3.13.0 with the options “—meta or —careful, -k 55,77,99,111,121” [47] was used to assemble high-quality reads into contigs. The contigs were binned into MAGs using the Binning module in MetaWRAP including metabat2 [48], maxbin2 [49] and concoct [50]. The reconstructed MAGs were further refined using the Bin refinement module in MetaWRAP. The gene prediction and annotation of contigs and the near-complete genome were initially annotated using Prokka [51]. To estimate the relative abundances of MAGs, normalized read coverage values were calculated using the Quant bins module in MetaWRAP.

For phylogenomic analysis, we selected MAGs with completeness of >50% and contamination of <10% (Supplementary Table 8). The taxonomic classification was performed according to the Genome Taxonomy Database (GTDB) taxonomy [52]. 16S rRNA gene sequences in MAGs were taxonomically classified according to SILVA ver. 138 [53]. Based on 120 concatenated single-copy marker proteins, a maximum likelihood tree was constructed for Pacearchaeota genomes. The GTDB-Tk v2.0.0 with the database version R207 was used to align and trim the concatenated protein sequences^77^. The resulting alignment was trimmed using trim-al v1.4.1 [54] with the “-gappyout” option. The maximum likelihood phylogenetic tree was generated using W-IQ-TREE [55] with 1000 replicates of ultrafast bootstrap [56] and visualized using Figtree v1.4.4 (http://tree.bio.ed.ac.uk/software/figtree/). The best fit model for the maximum likelihood phylogenetic tree was chosen automatically by W-IQ-TREE.

The Kyoto Encyclopedia of Genes and Genomes (KEGG) pathway tool [57], along with the BlastKOALA tool [58], was used for functional protein characterizations. We also used the KEGG Decoder to annotate protein sequences [59]. Curated sets of genes involved in hydrogen, carbon, nitrogen, and sulfur metabolism were searched using METABOLIC v.4.0 [60]. In addition to KEGG, TIGRfam [61], and Pfam [62], METABOLIC v.4.0 annotates genes using custom hidden Markov model profiles constructed for the software.

Metaproteomic analysis. The powdered chimney subsample was incubated in 12 mM SDC/12-mM SLS/100-mM TEAB buffer (pH 8.5) containing a 1% protease inhibitor (Protease Inhibitor Cocktail for General Use, Nacalai Tesque, Inc., Kyoto, Japan). After three cycles of sonication (30 s intervals, 0.5 s pulse on/0.5 s pulse off), the aliquot was cooled on ice. After centrifugation at 13000 × g for 3 min, the extracted proteins in lysis buffer (12 mM sodium deoxycholate, 12 mM sodium N-dodecanoylsarcosinate, and 50 mM ammonium bicarbonate containing 1% protease inhibitor cocktail for general use [Nacalai Tesque, Kyoto, Japan]) were reduced using 10 mM dithiothreitol at 37°C for 30 min following alkylation using 50 mM iodoacetamide at 37°C for 30 min in the dark. After five-fold dilution with 50 mM ammonium bicarbonate, the sample was digested using Lys-C (Wako, Osaka, Japan) at 37°C for 3 h following trypsin (Promega, Madison, WI, United States) at 37°C for 16 h. The digest acidified using trifluoroacetic acid was centrifugated at 15000 × g for 1 min, the supernatant of which was desalted with C18-StageTips [63] and dried under reduced pressure.

An ultra-high-performance nano-flow chromatography system and a hybrid trapped ion mobility spectrometry–quadrupole time of flight mass spectrometry system equipped with a nanoElute and a timsTOFPro (Bruker Daltonics, Bremen, Germany) were used for proteomic analysis. The dried sample was dissolved in formic acid (FA)/acetonitrile (ACN)/water (0.1/2/98, v/v/v) and then injected into a self-packed column (ACQUITY UPLC BEH C18, 1.7 μm, ID = 75 μm, length = 250 mm). For peptide separation, (A) FA/water (0.1/100, v/v) and (B) FA/ACN (0.1/100, v/v) were used at 60°C as the mobile phase. The composition of the mobile phase (B) was changed at 2%–35% for 100 min, 35%–80% for 10 min, and 80%–80% for 10 min, maintaining a flow rate of 280 nl/min. The eluted peptides were analyzed using a parallel accumulation serial fragmentation scan mode [64].

FragPipe (version 17.1) software was used to analyze liquid chromatography-mass spectrometry raw data [65]. Protein sequences of the MAGs reconstructed in this study (Supplementary Table 8) and 43 protein sequences considered contaminants (cRAP protein sequences [thegpm.org]) were used as reference databases. The enzyme was set to trypsin as a specific cleavage, and up to two missed cleavages were allowed in the proteolysis process. The allowed peptide lengths and mass ranges were 7–50 residues and 500–5000 Da, respectively. Carbamidomethylation at cysteine residues was set as a fixed modification, whereas N-acetylation at the protein N-terminus and oxidation at methionine residues were set as variable modifications, allowing for up to three sites per peptide. The peptide spectrum matches and the identified peptides/proteins were determined at <1% false discovery rage at the protein level.

## Results and discussion

### Physicochemical properties of the chimney interior

The inactive chimney was collected using the remotely operated vehicle Hyper-Dolphin at the Pika site [66]. The water depth and temperature of the sampling point were 2787 m and 1.7°C, respectively. Microscopic observations of a 20-μm-thick section of the inner chimney wall and subsequent image analysis revealed that the chalcopyrite grain boundaries have porosities of 15%–46% with an average pore throat radius of 2–5.3 μm in the inner chimney wall (Supplementary Fig. 1). Estimated permeabilities of 1 × 10^−15^–6 × 10^−14^ m^2^ fall within the range of sandstone [67] which potentially renders O_2_ into the chimney wall from seawater through grain boundaries. However, this is not the case for the inner chimney wall, given our previous data showing that cuprite (Cu_2_O), a mineral readily dissolved in contact with oxygenated seawater, is formed in the grain boundaries [33].

**Figure 1:**
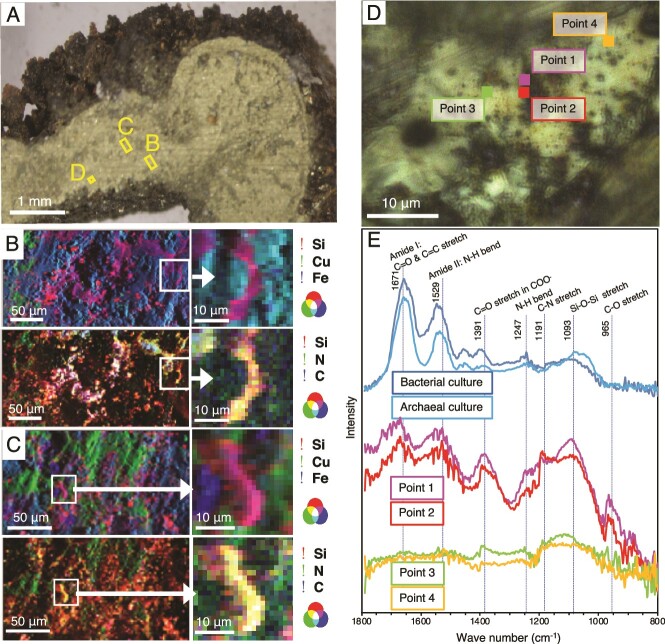
Submicron-scale spectroscopic analyses of chalcopyrite grain boundaries. A) A photograph of the inner chimney wall after cutting with a precision diamond wire saw. B, C) Elemental mapping of chalcopyrite grain boundaries obtained by synchrotron-based X-ray fluorescence analysis (XRF) from the regions with yellow rectangles in a. RGB diagrams are shown by synthesizing the three colors. D) A photograph of chalcopyrite grain boundaries with points analyzed by an infrared (IR) microscope. E) Optical photothermal infrared (O-PTIR) spectra of the points in d and cultured cells of *Nanobdella aerobiophila* strain MJ1^T^ (= JCM33616^T^) and *Metallosphaera sedula* strain MJ1HA (=JCM33617) for an archaeal reference and *Shewanella oneidensis* strain MR-1^T^ (=ATCC 700550^T^) for a bacterial reference. Peak assignment was based on refs [68–71] and references therein.

To clarify whether the chalcopyrite grain boundaries are not highly permeable because of mineral deposition, synchrotron-based scanning fluorescence X-ray microscopy was used to visualize the distributions of elements such as C, N, O, Al, Si, Fe, and Cu with a soft X-ray beam in a diameter range of 0.6–0.8 μm (Fig. 1A). It was revealed that the chalcopyrite grain boundaries in the inner and central regions of the inner chimney wall are primarily filled with SiO_2_ (Figs. 1B and C and Supplementary Fig. 2). It was also revealed that the high densities of C and N are distributed along the SiO_2_-infilling grain boundaries of chalcopyrite. Thus, we confirmed that the chimney wall is less permeable than the grain boundaries of chalcopyrite without the infilling of silica and C- and N-bearing materials.

The coexistence of microbial cells and SiO_2_ within the chalcopyrite grain boundaries was investigated by infrared (IR) spectroscopy at a submicron resolution [68]. IR spectra included peaks diagnostic of microbial cells (amide I and II [69]) and SiO_2_ (Si-O-Si stretch [70]) around a chalcopyrite grain (Figs. 1D and E). In addition, a peak attributed to carboxylic groups at ~1400 cm^−1^ was prominent in the spectra around the chalcopyrite grain, indicating the presence of extracellular polymeric substances commonly found in biofilms [71]. In situ spectroscopic analyses demonstrated the coexistence of microbial cells and SiO_2_ in the grain boundaries.

### High proportion of DPANN in the inner chimney wall

**Figure 2:**
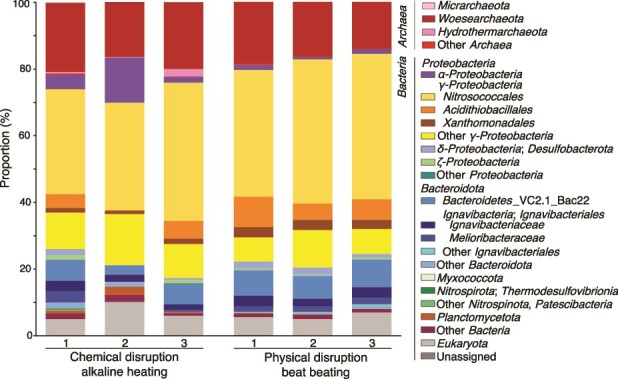
Microbial community structures from the chimney interior based on relative abundances by 16S rRNA gene sequences. Chemically and physically disrupted DNA extractants were analyzed for 16S rRNA gene amplicon sequences. The phylum and class were classified with the nomenclature based on the SILVA 138 database using QIIME2 software.

The bulk cell density of the inner chimney wall (2.3 × 10^8^ cells/cm^3^) was as high as those in metal sulfide chimneys from the Central Indian Ridge and the western Pacific [26]. Before performing metagenomic analysis, two DNA extraction methods, respectively, based on physical (beads beating) and chemical (alkaline heating) disruption methods were evaluated by 16S rRNA gene amplicon analysis for the chimney interior sample. The high proportion of DPANN was confirmed, regardless of the methods used (Fig. 2 and Supplementary Table 1). Phylogenetic analysis of DPANN-affiliated 16S rRNA gene sequences classified the DPANN as Woesearchaeota.

### Genomic features of the chimney *Pacearchaeota*

**Figure 3:**
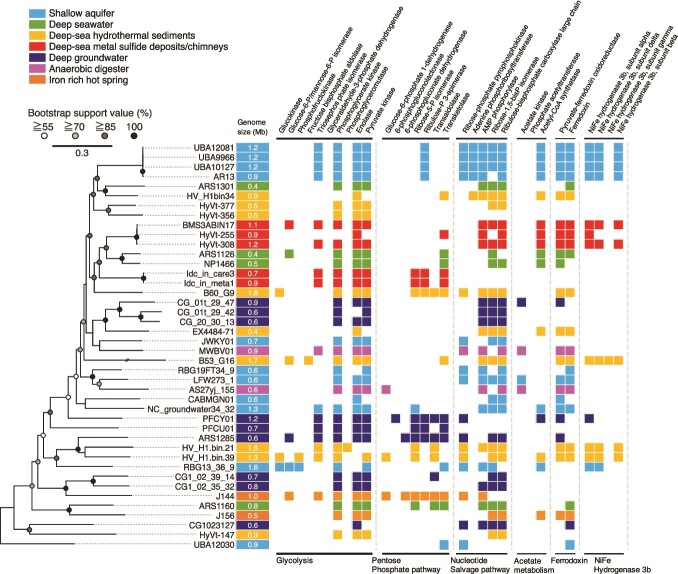
Comparative analysis of Pacearchaeota-affiliated genomes from various habitats. On the left, a maximum likelihood phylogenetic tree of *Pacearchaeota* MAGs from this study (Idc_in_care_mg3 and Idc_in_meta_mg8) and from public databases was constructed using 122 archaeal marker genes with three *Woesearchaeota* MAGs (not shown in the phylogenetic tree) as an outgroup (GW2011-AR15: GCA_000830295.1; GW2011-AR11:GCA_013330825.1; GW2011-AR9: GCA_013331635.1). The best fit model was selected as K2P + I + G4. On the right, an overview of the presence or absence of metabolic genes commonly found in *Pacearchaeota* MAGs is shown. Gene annotation was performed using BlastKOALA with default thresholds (see methods).

As the physically disrupted DNA extract is less damaged than that chemically disrupted, the former was subjected to genome-resolved metagenomic analysis. From the inner wall sample, near-complete genomes were reconstructed by several binning protocols. By the phylogenetic analysis of 16 rRNA gene sequences in the reconstructed genomes and from the amplicon libraries, the reconstructed genomes named Idc_in_care_mg3 and Idc_in_meta_mg8 (“care” and “meta” indicate the options for assembling high-quality reads into contigs) contained the 16S rRNA gene sequences nearly identical to the dominant DPANN lineage obtained by the amplicon sequence analysis (Supplementary Fig. 3 and Supplementary Table 2). The construction of a 122-concatenated-protein phylogenetic tree using the reconstructed genomes further confirmed the phylogenetic affiliation within *Pacearchaeota* rather than *Woesearchaeota* (Fig. 3) [72]. Based on 52 single-copy genes (Supplementary Tables 3) [73], the sizes of the reconstructed genomes were 0.72 and 0.88 Mbp, whereas their completeness were 84.6% and 92.3%, respectively (Supplementary Table 2).

In the *Pacearcaeota* genomes, nearly full sets of genes involved in glycolysis and the non-oxidative pentose phosphate pathway (PPP) were encoded, indicating adenosine triphosphate (ATP) generation via fermentation of sugar-based biomolecules (Fig. 3 and Supplementary Table 4). Many genes involved in nucleotide biosynthesis, particularly pyrimidine bases, were present in the chimney *Pacearchaeota* genomes (Supplementary Table 5). In contrast, genes involved in the biosynthesis of amino acids, lipids, and cofactors were absent. Furthermore, metabolic capabilities other than carbon metabolism are lacking (Supplementary Table 6). The limited biosynthetic capabilities indicate the nutritional dependence of the chimney *Pacearchaeota* on biosynthetic products from other organisms.

### Comparison of the *Pacearchaeota* genomes from various habitats

Although *Pacearchaeota* is detected from various ecosystems, its rarity and lack of obvious symbiotic partners make the lifestyle of *Pacearchaeota* enigmatic. As *Pacearchaeota* lineages across habitats and the genomic signatures of transitions among habitats remain unclear, we expand the inventory for *Pacearchaeota* genomes from natural and engineered environments such as anaerobic digesters [74, 75], shallow and deep aquifers [13, 14, 76, 77], iron-rich hot spring [78], deep seawater [79], deep-sea hydrothermal sediments [22, 80, 81], legacy radioactive waste trench water [82], sub-seafloor metal sulfide deposits [21], and deep-sea hydrothermal vent chimneys (Supplementary Table 2).

Moderate to high-quality genomes with completeness higher than 60% were profiled with respect to biosynthetic and other metabolic capacities, as well as basic genomic features (Fig. 3 and Supplementary Tables 4–6). The 122-concatenated-protein phylogenetic tree of the *Pacearchaeota* genomes shows that the phylogenomic relationships were related neither to habitat type nor genome size, which was common in the 16S rRNA gene tree (Supplementary Fig. 3). Their metabolic capacities are also not correlated with habitat types. The deep-sea metal sulfide deposits/chimneys hosted two distinct lineages with relatively large genome sizes (0.7–1.2 Mbp; Supplementary Table 2): one comprised the chimney genomes Idc_in_care_mg3 and Idc_in_meta_mg8, and the other comprised genomes from metal sulfide deposits in the same deep-sea hydrothermal field (BMS3ABIN17, HyVt-255, and HyVt-308). The two lineages were clustered with deep-sea *Pacearchaeota* lineages with small genomes (ARS1126 and NP1466) with high bootstrap values (96.6% and 87.3%). The lineage from the metal sulfide deposits appears to be metabolically flexible, based on the presence of genes involved in the nucleotide salvage pathway, Ni-Fe hydrogenase group 3b, and amino acid biosynthesis (Fig. 3 and Supplementary Tables 4–6).

### Genome annotations for the whole community in the chimney interior

To elucidate the biological interactions of *Pacearchaeota* with other co-occurring microbes, MAGs were constructed (Supplementary Tables 8 and 9). We found that MAGs with completeness higher than 50% and contamination lower than 10% were suitable to cover the phylogenetic diversity of co-occurring microbes. In addition to *Pacearchaeota*-affiliated genomes, we obtained nine taxonomically distinct genomes, five of which contained 16S rRNA gene sequences. As with *Pacearchaeota*, *Patescibacteria* (Idc_in_care_mg2 and Idc_in_meta_mg7) had genes involved in biosynthesis of pyrimidine bases and lacked genes for the biosynthesis of amino acids, lipids, and cofactors and other metabolic capacities (Supplementary Tables 10–12) [2, 5]. We found that *Nitrosococcaceae* (*Gammaproteobacteria*), the most abundant taxonomic group based on 16S rRNA gene amplicon sequences, was not found in MAGs (Supplementary Table 8). However, the *Nitrosococcaceae* sequences were expected to be taxonomically identical to gammaproteobacterial genomes classified as the order 21–64-14 without 16S rRNA gene sequences (Idc_in_meta_mg8 and 12). Hence, we constructed a phylogenetic tree based on 16S rRNA gene sequences from near-complete genomes of the order 21–64-14 in public databases and the *Nitrosococcaceae*-affiliated amplicon sequences (Supplementary Fig. 4 and Supplementary Table 13). As a result, the *Nitrosococcaceae* sequences were nearly identical to those from the order 21–64-14. Thus, the 21–64-14-affiliated genomes were confirmed to represent the abundant populations in the chimney interior.

### Metaproteomics resolved community-wide metabolic activities

**Figure 4:**
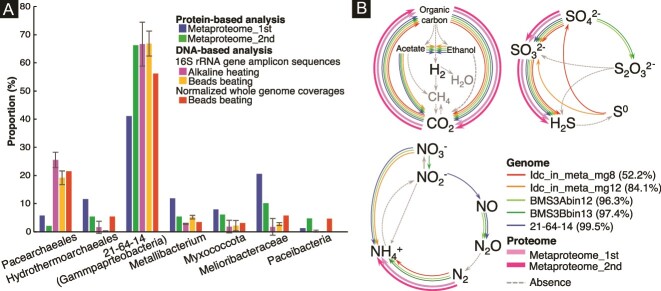
Taxonomic abundances and metabolic profiles of the chimney community. A) Comparison of taxonomic abundances based on protein- and DNA-based analyses. Proportions were obtained using selected data shown in Supplementary Tables 1 (16S rRNA gene amplicon sequences), eight (normalized whole genome coverages), 14, and 15 (proteomics). B) Metabolic pathways/genes involved in biogeochemical cycles of C, N, and S found in the genomic and proteomic inventories of 21–64-14-affiliated bacteria. A percentage in parentheses indicates genome completeness.

To understand the metabolic pathways and nutrient cycles potentially operated in the chimney interior, we annotated genes in the near-complete genomes from the dominant members of the whole community (Supplementary Tables 10–12). To constrain the partners of Pacearchaeota, it is crucial to clarify metabolically active members by metaproteomics. Duplicated extraction and sequencing led to 249 and 315 protein sequences, among which 133 and 219 protein sequences were obtained from the 21–64-14-affiliated genomes (Supplementary Tables 14 and 15). Relative abundances of the 21–64-14-affiliated genomes estimated by normalized genome coverages were similar to those estimated by 16S rRNA gene amplicon sequences and metaproteomic sequences (Fig. 4a). The 21–64-14-affiliated genomes included genes encoding a sulfate adenylyl transferase gene (*sat*), and anadenylyl-sulfate reductase genes (*aprAB*), and an array of dissimilatory sulfite reductase (*dsr*) genes. *dsrC* is involved in the reaction with *dsrAB* and sulfite and thought to be reduced by *dsrMK*(*JOP*) [83–85]. The presence of *sat*, *aprAB*, and *dsr* genes in the genome is known for dissimilatory sulfate reduction and sulfur oxidation. Sulfur oxidation is typically mediated by microbes, including *dsrEFH* for reactions with *dsrC* [86, 87]. The presence of *dsrMK*(*JOP*) and the absence of *dsrEF* support the inference that the 21–64-14-affiliated bacteria mediate dissimilatory sulfate reduction (Supplementary Tables 12). Metaproteomic analysis revealed that all genes involved in dissimilatory sulfate reduction, except for *dsrM* and *dsrP*, were detected in the chimney interior (Supplementary Table 16). In addition, metaproteomic detection of key enzymes for fixing carbon and nitrogen, such as Form I ribulose bisphosphate carboxylase/oxygenase (RuBisCO) large subunit, nitrogenase molybdenum-iron protein alpha chain (*nifD*), and nitrogenase iron protein (*nifH*) revealed the active primary production by the 21–64-14 bacteria (Fig. 4b and Supplementary Table 16). Metaproteomic analysis demonstrated the ecological importance of the order 21–64-14.

In contrast to the major occurrence of *Pacearchaeota* in the 16S rRNA gene amplicon sequence libraries and normalized whole genome coverages, *Pacearchaeota*-affiliated sequences were minor in the metaproteomic libraries (Fig. 4a). This difference may be reasonable, considering the small genome sizes of Pacearchaeota. *Pacearchaeota*-affiliated protein sequences were annotated as hypothetical proteins, translation initiation factor IF-2 subunit alpha, and pyruvate kinase (Supplementary Tables 14 and 15). Pyruvate kinase catalyzes the last step of glycolysis to produce ATP and pyruvate from AMP, phosphoenolpyruvate, and phosphate for energy conservation [88]. The minor occurrence of *Hydrothermarchaeales* in the 16S rRNA gene amplicon sequence libraries and normalized whole genome coverages was inconsistent with the frequent occurrence of *Hydrothermarchaeales*-affiliated sequences in the duplicated metaproteomic libraries (Fig. 4a). Both proteomic libraries included *Hydrothermarchaeales*-affiliated sequences annotated as archaeal chaperonin known to catalyze protein folding at high temperatures [89] and enolase involved in the last second step of glycolysis [90].

### Microbial succession across the termination of fluid venting

The sampling site is associated with the venting of black smokers (>300°C) [66]. The inner wall of chalcopyrite is universally formed in deep-sea hydrothermal vent chimneys, because the solubility of chalcopyrite decreases significantly at <300°C [91]. Although chalcopyrite grain boundaries are spatially available for microbes in the inner chimney wall, the temperature range of black smokers inhibits microbial colonization. After the transition from high- to low-temperature fluids, chemolithotrophic microbes appear to be hosted in the chalcopyrite grain boundaries. After the cessation of fluid venting, the cold chimney interior is limited to energy sources from fluids.

**Figure 5:**
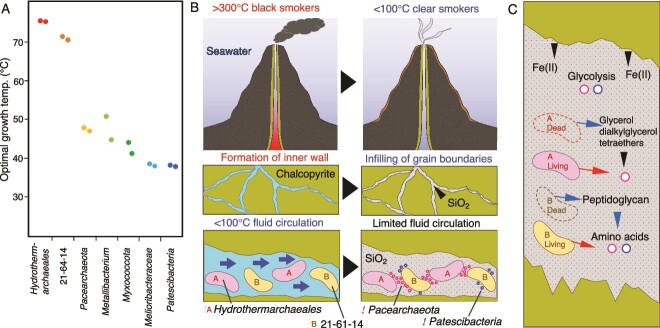
Temperature dependence of mineral formation and microbial succession in a deep-sea hydrothermal vent chimney. A) Optimal growth temperatures of abundant members of the chimney community predicted by the frequencies of seven amino acids (IVYWREL). B) Schematic illustrations of fluid venting, mineral formation, and key microbial populations. The blue arrows indicate the flow of low-temperature fluids. C) Schematic illustration of the transfer of chemicals in the mineral environment. Black, blue and red arrows indicate the transfers from minerals, dead cells and living cells.

To distinguish microbial populations energetically depending on low-temperature fluids, the optimal growth temperature (OPT) was estimated for the chimney community based on the frequencies of the seven amino acids Ile, Val, Tyr, Trp, Arg, Glu, and Leu (IVYWREL) in whole genome sequences [92] (Fig. 5A and Supplementary Table 8). The OPT of *Hydorothermarchaeales* was ~70°C, consistent with the dominance of *Hydorothermarchaeales* in Juan de Fuca Ridge flank crustal fluids at 67°C [93]. Unexpectedly, the 21–64-14-affiliated genomes from the inner wall sample had high frequencies of IVYWREL (estimated OPTs of ~67°C), as well as some representative genomes of the 21–64-14 order (Supplementary Table 13). The low OPTs of *Pacearchaeota* (~44°C) and *Patescibacteria* (35°C) indicate the later colonization after the termination of fluid venting. A recent metagenomic study of bulk chimney samples with various formation ages clarified that *Nitrospirae* becomes predominant during several years after the termination of fluid venting [25]. In contrast, *Nitrospirae* disappears in chimney samples with formation ages of several thousand years. The outer porous wall of our chimney sample is dominantly colonized by *Nitrospirae* [34], which is consistent with the recent termination of fluid venting. Given the preservation of the fine tips of the sampled chimney [33], the timescale of venting cessation might be years to decades. In this study, microbial succussion represented by *Hydorothermarchaeales* and the 21–64-14 order to *Pacearchaeota* and *Patescibacteria* is uniquely described in the inner massive wall.

Environmental factors controlling microbial habitability in the inner chimney wall.

Given that metaproteogenomic data were obtained from the inner wall separated from the outer porous layers, metabolic activities based on protein expression are derived from microbes thriving in the inner chimney wall. Thus, it is critical to clarify the microbial distribution within the inner massive wall. Although the IR spectroscopy can detect single microbial cells, the limit of detection of C and N by synchrotron-based XRF analysis is ~1000 ppm [39]. Thus, the high cell densities are localized along the grain boundaries of chalcopyrite. The combination of the advanced spectroscopic techniques is powerful, because the distribution of inorganic solid material is simultaneously obtained with microbial distribution at the submicron scale. In this study, the co-distribution of silica and microbial cells was unambiguously observed from large to small regions in the inner massive wall.

Our previous study showed the dense colonization of ultrasmall cells (~100 nm in cell diameter) in the silica-deposited pores [33]. Although our microscopic techniques are incapable of distinguishing small cells between *Pacearchaeota* and *Patescibacteria*, it is suggested from the normalized read coverages of their genome bins that *Pacearchaeota* is more abundant than *Patescibacteria* (Fig. 4A and Supplementary Table 8). The silica deposition along the chalcopyrite grain boundaries substantially reduces the transport of large cells. In addition, the reduction of O_2_ penetration through the chalcopyrite grain boundaries by the silica deposition favors the anaerobic metabolism of *Pacearchaeota*. Particularly, glycolysis and the PPP, the central metabolism of the Chimney *Pacearchaeota* revealed by genome-resolved omics, are catalyzed by Fe(II) without enzymatic activities [94, 95]. Given the Fe(II) availability from the dissolution of chalcopyrite for non-enzymatic reactions, the chimney *Pacearchaeota* has some advantage in reducing protein expression.

Biological interactions of *Pacearchaeota* with co-occurring microbes.

*Pacearchaeota* with the smallest median genome size across DPANN probably adopts a nutritional strategy depending on co-existing microbes, given their highly reduced biosynthetic and metabolic capacities [2, 5]. However, microorganisms associated with *Pacearcheota* remain unknown, primarily because of the minor occurrence of *Pacearchaeota* in diverse microbial communities. The abundance of *Pacearchaeota* associated with metabolically active, taxonomically limited groups has some advantage in providing nutritional insights. FISH analysis targeting *Archaea* and *Pacearchaeota* has been previously performed for thin sections of the inner chimney wall to correlate the spatial distribution of *Pacearchaeota* and the co-existing microbial microorganisms in the grain boundaries [33]. Although microbial cells in the grain boundaries were visualized using SYBR-Green I staining, hybridization signals neither from archaea-universal nor *Pacearchaeota*-specific probes are evident. It is therefore impossible to clarify the physical contact between *Pacearchaeota* and the co-existing archaea.

As many *Pacearchaeota* genomes include genes encoding murein transglycosylase (GH23 in the CAZy database) that specifically binds and degrades peptidoglycan, a symbiotic relationship with bacteria is speculated [96]. Although murein transglycosylase was absent in the chimney *Pacearchaeota* genomes (Supplementary Table 17), muramoyltetrapeptide carboxypeptidase, which releases alanine from peptidoglycan [96], was found in abundance in the *Pacearchaeota* genomes from the chimney and various habitats (Supplementary Table 7). Metaproteomic evidence of bacterial primary production in the chimney interior agrees with the supply of bacterial amino acids to *Pacearcheota*. The successful enrichment of DPANN only with bacterial growth from deep-sea hydrothermal vent fluids also supports this possibility [19].

The isoprenoid biosynthesis genes for archaeal mevalonate were present in the *Hydrothermarchaeales* genomes (Supplementary Table 10), indicating the supply of archaeal lipids from *Hydrothermarchaeales* to *Pacearchaeota*. All *Hydrothermarchaeales* genomes, including the chimney *Hydrothermarchaeales*, encode genes for chemolithoautotrophy via the Wood Ljungdahl pathway and for the usage of CO and H_2_ as electron donors [93, 97]. Given that CO and H_2_ are enriched in hydrothermal fluids, the cellular maintenance rather than the active incorporation of inorganic carbon for the growth suggested through our metaproteomic analysis is consistent with the metabolic shift after the termination of fluid venting. At terrestrial hot springs, archaeal lipids such as glycerol dialkylglycerol tetraethers are retained in silica sinters after the cessation of fluid venting [98]. After the death of *Hydrothermarchaeales*, archaeal lipids released from *Hydrothermarchaeales* cells and bound to silica could be utilized by *Pacearchaeota*.

Given that alkaline heating used for the chemical disruption method substantially accelerates the dissolution of amorphous silica [40], high proportions of *Pacearchaeota* and *Hydrothermarchales* obtained by 16S rRNA gene amplicon analysis of DNA extracted by the chemical disruption method than those extracted by the physical disruption method (bead beating) could be explained by the release of *Pacearchaeota* and *Hydrothermarchales* cells and/or their DNA entrapped within amorphous silica. This result supports the spatial association of *Pacearchaeota* and *Hydrothermarchales* with the silica deposit within the chalcopyrite grain boundaries.

Based on phylogenetic similarity between homologous genes in genomes, horizontal gene transfer (HGT) has been previously described for DPANN symbionts and their hosts [9, 10]. HGT between the genomes of *Pacearcheota* and *Hydrothermarchaeales* was therefore investigated for the indirect evidence of symbiosis*.* To detect horizontal gene transfer between the genomes of *Pacearcheota* and *Hydrothermarchaeales*, we performed homology search using BLASTp for the protein sequences of all genes of the *Pacearcheota* MAGs as queries against a custom database including the *Hydrothermarchaeales* genomes and over 400 representative archaeal complete genomes (Supplementary Table 18). For the *Pacearcheota* genes close to the *Hydrothermarchaeales* genes, phylogenetic trees were constructed with similar sequences of top 500 hits by BLASTp against the NCBI nr database. As a result, no genes of the *Pacearcheota* were clustered with the *Hydrothermarchaeales*. Thus, it is concluded that no HGT occurred between the *Pacearcheota* and *Hydrothermarchaeales* in the chimney sample. In addition, the difference in OPT between *Hydorothermarchaeales* and *Pacearchaeota* seems to be too significant to form the symbiotic relationship typically found in other DPANN archaea [9, 10]. Taken all together, it is likely that free-living *Pacearcheota* with the minimized biosynthetic capacity depends on metabolites produced by the co-occurring microbes in the chimney community.

### Ecological implications for rock-hosted DPANN archaea

Amorphous silica is ubiquitously formed inside the chimney during the transition from high- to low-temperature fluid venting (Fig. 5B) [99]. Thus, the colonization of the thermophilic populations is accompanied by the deposition of amorphous silica in chalcopyrite grain boundaries. After the termination of fluid venting, the growth of *Pacearhaeota* is favorable in the chalcopyrite grain boundaries, because of the co-existence of amorphous silica and thermophilic chemoautotrophs (Fig. 5B). It is likely that amino acids and lipids are supplied to *Pacearchaeota* directly from living organisms (red arrows in Fig. 5C). In the mineral environment, the supply of Fe(II) that catalyzes abiotic glycolysis reactions may be important for the persistence of *Pacearhaeota* (Fig. 5C). Glycerol dialkylglycerol tetraethers retained by silica after the death of *Hydrothermoarchaeales* might be utilized by *Pacearchaeota*. Peptidoglycan in dead bacterial cells could be an important source of amino acids (Fig. 5C). *Patescibacteria* are known to share many traits, including small cell size, metabolic deficiency, and an epibiotic lifestyle [2, 5]. Glycolysis-related genes found in the *Patescibacteria* genomes suggest they may take advantage of the chalcopyrite grain boundaries (Fig. 5C; Supplementary Table 10).

Fe(II)-bearing minerals are ubiquitous in igneous and sedimentary rocks. Amorphous silica is universally formed where silicate minerals interact with water in host rocks [100]. Rock-hosted DPANN archaea collected from fluids might have the free-living lifestyle where chemoautotrophic biomass production occurs in pores and fractures filled with amorphous silica [101]. Future studies clarifying the relationship of DPANN archaea from fluids and rocks from the same habitat could shed light on the lifestyle of enigmatic DPANN archaea in the rocky biosphere.

## Supplementary Material

Takamiya_SupplementaryMaterial_wrae207

ISME_SupplementaryTables_wrae207

## Data Availability

All data required to evaluate the conclusions in the paper are present in the paper and/or the Supplementary Materials. The 16S rRNA gene sequences in this study were all deposited in the DDBJ nucleotide sequence database with accession numbers LC554901-LC555740. The MAG sequences were deposited under the BioProject accession number PRJDB13464.
