# Supplementary material for: Genome-resolved metaproteogenomic and nanosolid characterization of an inactive vent chimney densely colonized by enigmatic DPANN archaea
Source: ISME J. 2024 Nov 5;18(1):wrae207. doi: 10.1093/ismejo/wrae207 (PMC11537232; doi:10.1093/ismejo/wrae207)
Supplement: Takamiya_SupplementaryMaterial_wrae207 [file takamiya_supplementarymaterial_wrae207.docx]

**Supplementary Material**

**Genome-resolved metaproteogenomic and nanosolid characterization of an inactive vent chimney densely colonized by enigmatic DPANN archaea**

Hinako Takamiya^1†^, Mariko Kouduka^1†^, Shingo Kato^2^, Hiroki Suga^3^, Masaki Oura^4^, Tadashi Yokoyama^5^, Michio Suzuki^6^, Masaru Mori^7^, Akio Kanai^7^, and Yohey Suzuki^1^*

^1^Department of Earth and Planetary Science, The University of Tokyo, 7-3-1 Hongo, Bunkyo-ku, Tokyo 113-0033, Japan

^2^Japan Collection of Microorganisms (JCM), RIKEN BioResource Research Center, 3-1-1 Koyadai, Tsukuba, Ibaraki 305-0074, Japan

^3^Dynamic Spectroscopy and Imaging Team, Spectroscopy and Imaging Division, Japan Synchrotron Radiation Research Institute (JASRI), 1-1-1 Kouto, Sayo-gun, Hyogo, 679-5148, Japan

^4^Soft X-ray Spectroscopy Instrumentation Team, RIKEN SPring-8 Center, 1-1-1 Kouto, Sayo-gun, Hyogo, 679-5148, Japan

^5^Graduate School of Advanced Science and Engineering, Hiroshima University, 1-7-1 Kagamiyama, Higashi-Hiroshima, Hiroshima, Japan

^6^Department of Applied Biological Chemistry, The University of Tokyo, 1-1-1 Yayoi, Bunkyo-ku, Tokyo 113-8657, Japan

^7^Institute for Advanced Biosciences, Keio University, 403-1, Nihonkoku, Daihoji, Tsuruoka, Yamagata, 997-0017, Japan.

^†^These authors contributed equally: Hinako Takamiya, Mariko Kouduka

*Corresponding author. E-mail: yohey-suzuki@eps.s.u-tokyo.ac.jp (Y.S)


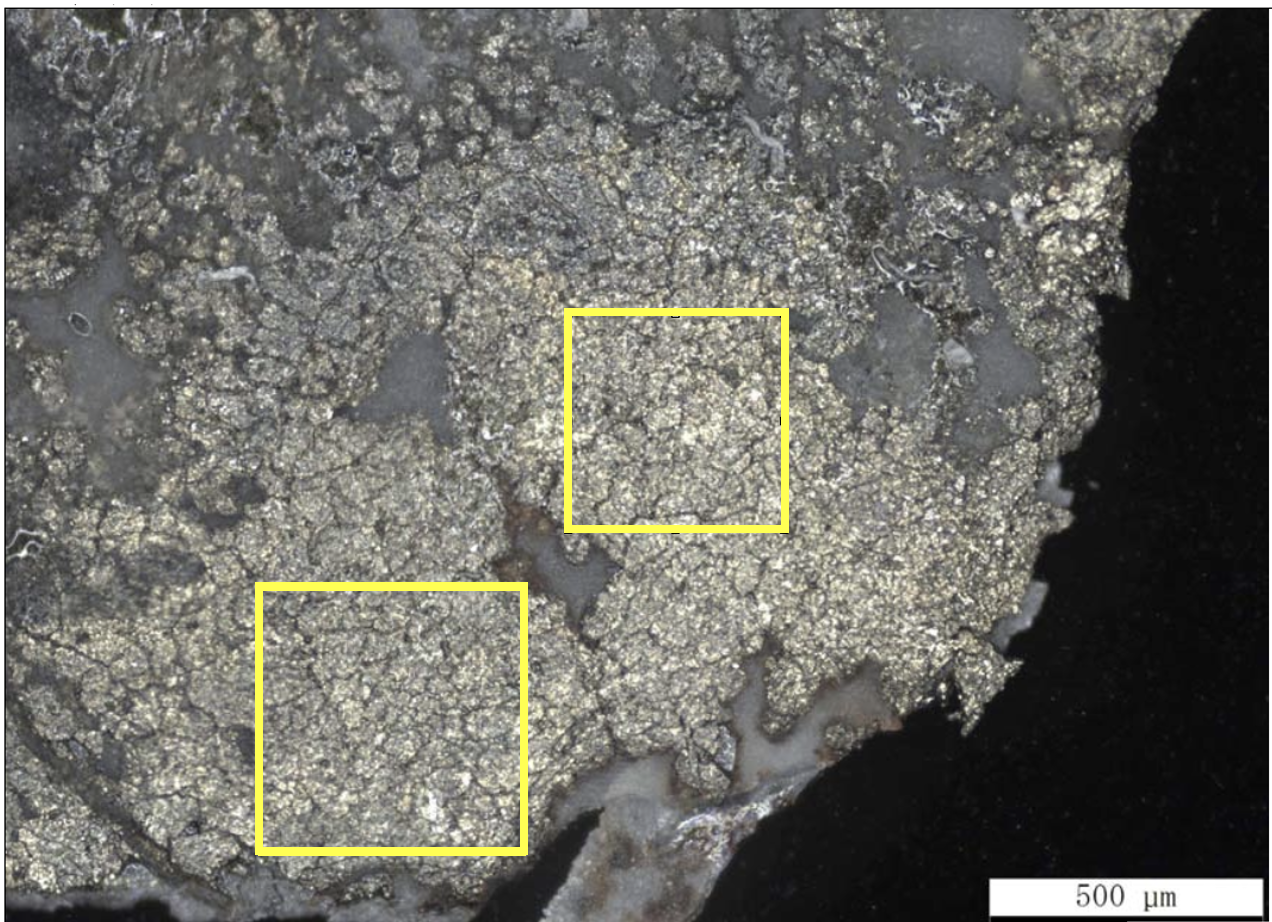


**Supplementary Fig. 1. Chalcopyrite grain boundaries analyzed for porosity and permeability.** A microscope image of a 20-μm-thick section from the inner chimney wall previously characterized [33]. The images in the yellow square were processed to measure porosity around chalcopyrite grains.


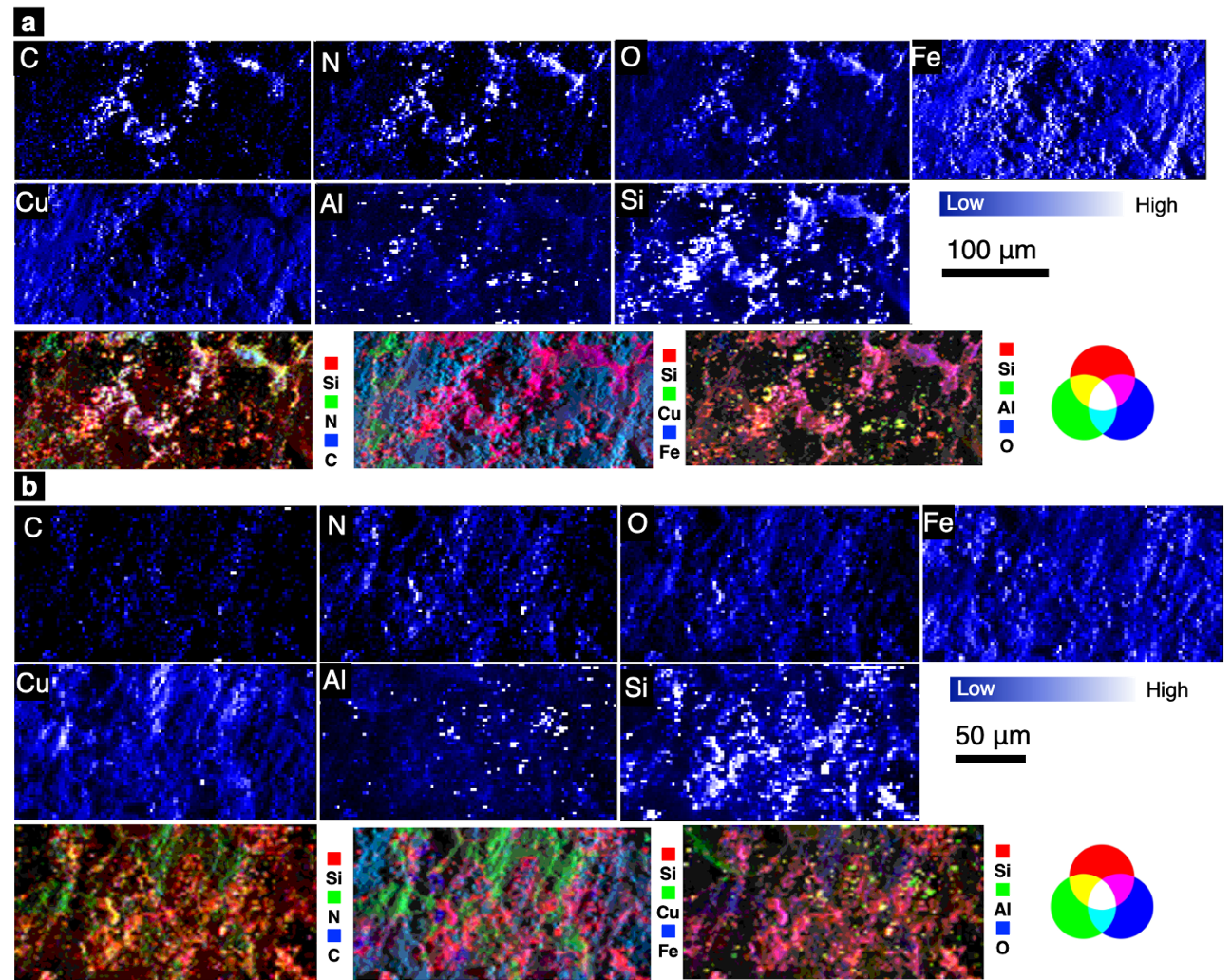


**Supplementary Fig. 2. Elemental distributions of chalcopyrite grain boundaries. a,b,** elemental maps obtained by synchrotron-based X-ray fluorescence analysis (XRF) with RGB color synthesis of selected elements from the regions with yellow rectangles labeled, respectively, with c and d in Fig 4a.

**Supplementary Fig. 3. Analysis of Pacearchaeota-affiliated 16S rRNA gene sequences from the chimney interior and public databases.** A maximum likelihood tree consists of 16S rRNA gene sequences from polymerase chain reaction (PCR) amplification with primers and from MAGs. The best fit model was selected as K2P+I+G4. 16S rRNA gene amplicon sequences obtained from the chimney interior in this study are prefixed with “Idc_int_,” whereas 16S rRNA gene sequences in MAGs are shown in bold text.

**Supplementary Fig. 4. 16S rRNA gene sequence analysis of Nitrosococcaceae- and 21-64-14-affiliated sequences.** A maximum likelihood tree consists of 16S rRNA gene sequences from PCR amplification with primers and from MAGs. The best fit model was selected as TIM2+F+I+G4. 16S rRNA gene amplicon sequences obtained from the chimney interior in this study are prefixed with “Idc_int_,” whereas 16S rRNA gene sequences in MAGs are shown in bold text.
